# The Importance of Vertical and Horizontal Dimensions of the Sediment Matrix in Structuring Nematodes Across Spatial Scales

**DOI:** 10.1371/journal.pone.0077704

**Published:** 2013-10-30

**Authors:** Danilo Cândido Vieira, Gustavo Fonseca

**Affiliations:** 1 Centro de Estudos do Mar (CEM), Universidade Federal do Paraná (UFPR), Pontal do Paraná, Paraná, Brazil; 2 Centro de Biologia Marinha (CEBIMar), Universidade de São Paulo (USP), São Sebastião, São Paulo, Brazil; National Institute of Water & Atmospheric Research, New Zealand

## Abstract

Intensive surveys have been conducted to unravel spatial patterns of benthic infauna communities. Although it has been recognized that benthic organisms are spatially structured along the horizontal and vertical dimensions of the sediment, little is known on how these two dimensions interact with each other. In this study we investigated the interdependence between the vertical and horizontal dimensions in structuring marine nematodes assemblages. We tested whether the similarity in nematode species composition along the horizontal dimension was dependent on the vertical layer of the sediment. To test this hypothesis, three-cm interval sediment samples (15 cm depth) were taken independently from two bedforms in three estuaries. Results indicated that assemblages living in the top layers are more abundant, species rich and less variable, in terms of species presence/absence and relative abundances, than assemblages living in the deeper layers. Results showed that redox potential explained the greatest amount (12%) of variability in species composition, more than depth or particle size. The fauna inhabiting the more oxygenated layers were more homogeneous across the horizontal scales than those from the reduced layers. In contrast to previous studies, which suggested that reduced layers are characterized by a specific set of tolerant species, the present study showed that species assemblages in the deeper layers are more causal (characterized mainly by vagrant species). The proposed mechanism is that at the superficial oxygenated layers, species have higher chances of being resuspended and displaced over longer distances by passive transport, while at the deeper anoxic layers they are restricted to active dispersal from the above and nearby sediments. Such restriction in the dispersal potential together with the unfavorable environmental conditions leads to randomness in the presence of species resulting in the high variability between assemblages along the horizontal dimension.

## Introduction

The sediment is a three-dimensional habitat for a vast number of infauna species. In the sediment, these benthic organisms are spatially structured through of a variety of environmental factors, such as granulometry, salinity and oxygen and food availability, along both the horizontal and vertical dimensions [1]–[3]. Specially for nematodes, the most abundant and species rich taxa of marine sediments [4], [5], at the horizontal dimension the variability of the fauna at the scale of centimeters is as large as at the scale of meters to hundreds of kilometers [6]–[8]. At the vertical dimension, changes in nematode community structure occur at the scale of few centimeter, due to a more pronounced change in environmental factors, such as food resources and oxygen availability [3], [9], [10]. These abrupt changes along the vertical dimension cause significant decreases in nematode densities, numbers of species and changes in species composition [3], [11], [12]. In fact, only few meiofaunal taxa can persist to extreme reduced conditions at the deeper layers, and although nematodes are considered a group very tolerant to such conditions, this tolerance is considered species-specific. [2], [13]. Reduced layers impose therefore a strong habitat selection for the fauna. Although the vertical pattern is already well established in the meiobenthic literature [3], [9], [14], [15], little has been done to understand how the vertical and horizontal patterns interact with each other. For instance, we still do not know whether superficial and deep dwelling species show similar spatial patterns at the horizontal scale. This lack of knowledge is, at least in part, consequence of the dependent sampling design traditionally used in infauna studies [3], [16]–[18]. In this design, vertical subsamples are taken from the same corer restricting thus the comparisons of vertical layers from multiple sites along horizontal scales.

Oxygen is recognized as a major structuring factor of metazoan communities in marine sediments, along both horizontal and vertical dimensions [19], [20]. The availability of oxygen to benthic system depends on the oxygen demand for organic matter degradation and on the supply through several transport mechanisms [19]. Surface sediments are generally more oxygenated than deeper sediments where oxi-reduction reactions predominate. The depth of the oxygenated layer is variable depending on the balance between hydrodynamic regime, bioturbation and organic degradation [21], [22]. For example, in high-energy environments, such as sandy beaches, the oxygenated layer can reach depths greater than 20 cm, because of the high drainage maintained by the strong hydrodynamics regime. Meanwhile, in low-energy environments, such as muddy estuarine sediments, the oxygenated layer is restricted to few millimeters, because a consequence of high organic loads and weak hydrodynamics regime [1], [23].

Based on the current understanding of the vertical distribution of the fauna and of the redox profile, it can be hypothesized that the species poor assemblages inhabiting the more reduced layers of the sediment will be characterized by few tolerant species. If this pattern proves to be consistent at multiple sites (horizontal scale), we can expect that the deep samples will have the same set of tolerant species and high similarity in the multivariate analysis. The species rich superficial assemblages in comparison would be expected to be more heterogeneous with the composition of species highly dependent on local disturbances and colonization rates that occur at random [24], [25].

In order to test the interdependence between the vertical and horizontal dimensions in structuring marine nematodes assemblages, this study analyzed the nematode vertical profile using an independent sampling design over multiple spatial scales. Estuarine bedforms were selected to test our hypothesis. Estuarine bedforms are sandy environments formed from patterns of sediment transport governed by hydrodynamic forces such as tidal currents, river discharge and wind driven currents. Given the high organic conditions of estuaries, reduced conditions are usually present at the deeper layers of the sediment [26].

## Materials and Methods

### 1 - Ethics Statement

No specific permits were required to collect these animals, because meiofauna are microscopic, non-pathogenic animals, field study did not involve endangered species and sampling was carried out in public estuaries. Moreover, no meiofauna species are under special conservation concerns.

### 2 - Study Area and Sampling Design

**Figure 1:**
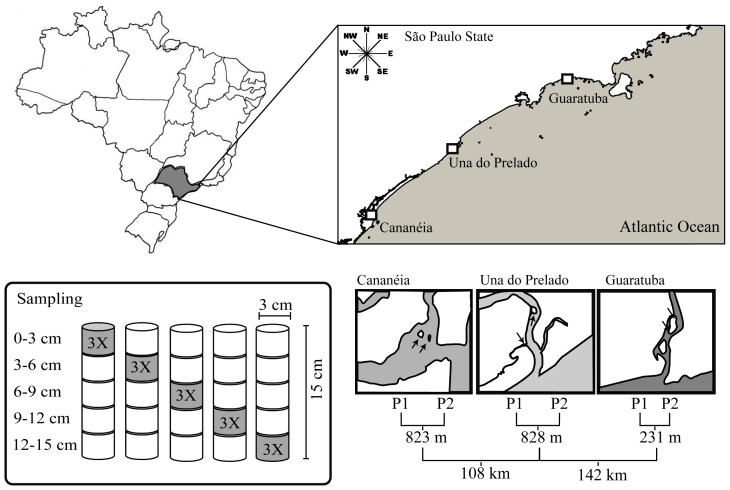
Map of the sampling sites and schematic view of the sampling design. Arrows represent the unvegetated bedforms. P1 and P2 represent the respective plots sampled in each estuary.

Three estuarine systems (Una do Prelado, Cananéia, Guaratuba) were sampled along the southeast coast of Brazil in February 2011 (Fig. 1). The region is influenced by a mean tidal range of 0.76 m. At all estuaries, bedforms were exposed and submerged at low and high tide, respectively.

A hierarchical sampling design was used to determine variation in community structure at 3 horizontal scales ranging from meters to hundreds of kilometers, and 5 vertical sediment layers (Figure 1). In each of the three estuaries two unvegetated bedforms (plots) were sampled (scale of hundreds of meters) when exposed during low tide and similar meteorological conditions. In all estuaries, sampling began 1 hour before peak ebb and ended 1 hour after. During sampling, no visual differences in terms of sediment surface (e.g. ripples formation, depressions, etc.) were observed between bedforms of the different estuaries. At each plot, random samples several meters apart from each other were collected with a corer (3 cm diameter, 15 cm length) to obtain meiofauna and redox potential measures. In order to analyze the vertical distribution of the meiofauna, five layers of sediment (0–3, 3–6, 6–9, 9–12, 12–15 cm), each from a different core, were sampled three times. In total, 90 samples were analyzed. The fauna samples were immediately fixed in 4% formaldehyde solution. Redox potential of the sediment was measured with electrodes inserted into the middle depth of each sediment layer after slicing. Two additional samples for analysis of organic matter and granulometry were obtained with a corer of 5 cm in diameter. In order to increase the volume of sediment collected for the granulometry analysis, each layer was collected three times. For logistical constraints, only one sample for organic matter and granulometry was taken.

### 3 - Sample Processing

Meiofauna samples were washed through a 45 µm sieve, extracted by flotation with a solution of colloidal silica (LUDOX TM-50) with density of 1.18 g.cm^−3^ [14]. The samples were stained with Rose Bengal and major taxonomic groups were counted under a stereomicroscope. 10% of the nematodes were randomly picked (randomly selecting squares in a gridded dish and taking out all specimens encountered at selected square until reach the desired number of individuals) for identification, unless densities were smaller than 120, then all individuals were identified. Nematodes were first transferred to anhydrous glycerol (5%) and then mounted on permanent slides. Nematodes were identified to genus level [5] and separated into morphospecies [27]. In addition we classified the nematodes according to the body shape: slender (length/width ratio >14 µm) or stout (length/width ratio <14 µm) [28].

Organic matter samples were dried at 80°C until reaching a constant weight. They were then re-weighed and organic material was combusted in a muffle furnace at 550°C for 4 h [29]. Wet, dry and ash-free dry weight values were used to calculate water content and organic content of the sediment through the difference between wet and dry weight and between dry and ash-free dry weight, respectively. Granulometric analysis was carried out using an automatic sieve shaker with different mesh sizes (1.000, 0.500, 0.125, 0.063 and 0.063 mm) for 20 min. The dry weight of each fraction was determined and the proportion that each fraction contributed to total mass was calculated [30]. Sediment statistical parameters were calculated using the SysGran v3 software [31].

### 4 - Data Analysis

For both univariate and multivariate data, the statistical design comprised the following factors: Estuary (Random), Plot nested within Estuary (Random) and Sediment Layer (Fixed). The interaction effect between the factors “Estuary*Layers” and “Plot(Estuary)*Layers” were also tested (Table 1).

**Table 1:** Summary of the ANOVA mixed models design used to analyze the data sets.

Source of variation	Abrev.	Type	Numerator	Denominator	Num.df
Estuary	E	R	1*E	1*P(E)	2
Layer	L	F	1*L	1*E x L	4
Plot(Estuary)	P(E)	R	1*P(E)	1*Res	3
Estuary* Layer	E*L		1*E x L	1*P(E) x L	8
Plot(Estuary)* Layer	P(E)*L		1*P(E) x La	1*Res	12

Abrev: abbreviation; Type of factor: Random (R) or Fixed (F); Respective numerator, denominator and degrees of freedom (df) used to calculate the F-ratios.

Abiotic data was analyzed by means of principal component ordination (PCA). Redox potential was analyzed by means of analysis of variance using mixed models design (mixed-ANOVA; Table 1). In order to differentiate the oxidation degree of the different layers of the sediment, redox potential values were separated in four classes: “strongly oxidized” (>100 mV), “oxidized” (0 mV<×<100 mV), “reduced” (0 mv<×<−100 mV) and “strongly reduced” sediments (<−100 mV) [32], [33].

As univariate descriptors of nematodes we used abundance (10 cm^2^), species richness and species diversity expressed as expected number of species after randomly sampling 21 (ES21) individuals. These data were also treated statistically by mixed-ANOVA (Table 1), preceded by Cochran’s test for homogeneity of variances. ANOVA was performed in the R environment with the aid package GAD [34]. *Posteriori* Student-Newman-Keuls (SNK) multiple comparisons tests were used to investigate differences among means.

The analytical design used for the multivariate analyses of the fauna was the same used for the univariate measures (Table 1). All tests were applied on a Bray–Curtis similarity matrix underlying the classification of samples (factors). Prior to the analysis, data were standardized and transformed when necessary. Since our data set was characterized by many samples having few individuals or even no individuals, increasing significantly the variability of the data, we added a dummy variable (weight 1) to the matrix [35].

Permutational multivariate analysis of variance - PERMANOVA [36] (based on data of presence/absence of species and Bray-Curtis similarity matrix) was used to analyze how the multivariate structure of the assemblage varied at the different spatial scales and sediment layers (Table 1). Multidimensional scaling (MDS) was used to visualize the relationships between samples. Since clusters were detected (see results sections), similarity percentage analysis procedure (SIMPER) was used as an explanatory analysis to indentify the set of species which were responsible for the cluster formation. A distance-based multivariate linear model (DistLM), using forward selection, was performed to determine how much each abiotic variable explained the total variation of the species composition (software PRIMER 6 & PERMANOVA). Highly correlated variables (r>0.9were omitted for the DistLM procedures. At the end, eight environmental variables were included in the regression analysis: Redox potential (mV), pore water (%), organic matter (%), skewness (sk - skewness of sediment grain size distribution), %sand, %silt, medium sand (g) and very fine sand (g). P–values were obtained using 999 permutations of the raw data.

The degree of variability in the composition and relative abundance of species in the community was assessed through permutational multivariate dispersion (PERMDISP, [37]) for each sediment layer at the three spatial scales: all estuaries together, within estuaries and within plots. To test whether the dispersion of the data varied according to the redox potential of the sediment, regression analyses were conducted for each spatial scale.

## Results

### 1 - Environmental Characterization

**Figure 2:**
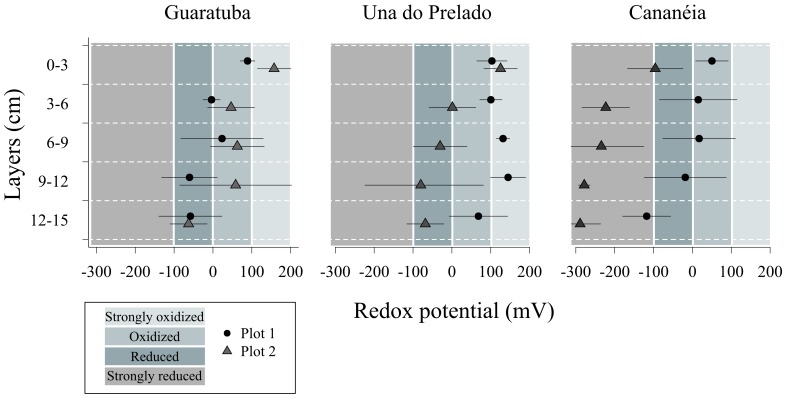
Vertical profile of redox potential values of the two bedforms sampled at each estuary. Bars represent standard deviation from the mean (n = 3 for each point).

No differences in redox potentials were found between estuaries (p>0.05; Table 2), whereas plots within estuaries and sediment layers differed significantly (p<0.05). At all estuaries redox potential decreased with increasing sediment depth (Figure 2). Post-hoc SNK-tests showed significant differences between L1 and L5, and no difference between the intermediate layers (L2, L3 and L4).

**Table 2:** Analysis of variance (ANOVA) and Permutational multivariate analysis of variance (PERMANOVA).

Variable	Source of variation	DF	MS	F	P
Redox potential	Estuary	2	244071.2	1.55	0.345
	Layer	4	60741.2	41.73	**0.000**
	Plot(Estuary)	3	157247.3	36.30	**0.000**
	Estuary*Layer	8	1455.5	0.22	0.980
	Plot(Estuary)*Layer	12	6538.4	1.51	0.146
	Residual	60	4332.0		
Richness species	Estuary	2	183.6	1.04	0.455
	Layer	4	90.6	4.26	**0.039**
	Plot(Estuary)	3	177.1	20.97	**0.000**
	Estuary*Layer	8	21.2	0.80	0.614
	Plot(Estuary)*Layer	12	26.5	3.14	**0.002**
	Residual	60	8.4		
ES 21	Estuary	2	183.6	1.03	0.455
	Layer	4	90.5	4.26	**0.038**
	Plot(Estuary)	3	177.1	20.97	**0.000**
	Estuary*Layer	8	21.2	0.80	0.614
	Plot(Estuary)*Layer	12	26.5	3.14	**0.002**
	Residual	60	8.4		
Abundance	Estuary	2	260.5	2.43	0.236
	Layer	4	297.8	17.04	**0.001**
	Plot(Estuary)	3	107.4	6.56	**0.001**
	Estuary*Layer	8	17.5	0.55	0.797
	Plot(Estuary)*Layer	12	31.7	1.94	**0.048**
	Residual	60			
PERMANOVA	Estuary	2	26246	1.63	0.06
*Community*	Layer	4	6050.4	3.32	**0.003**
	Plot(Estuary)	3	16079	17.08	**0.001**
	Estuary*Layer	8	1819.6	1.04	0.402
	Plot(Estuary)*Layer	12	1738.9	1.86	**0.001**
	Residual	60	942.2		

Bold lettering identifies those P values that are significant (<0.05). Df: degress of freedom; MS: mean squares.

**Figure 3:**
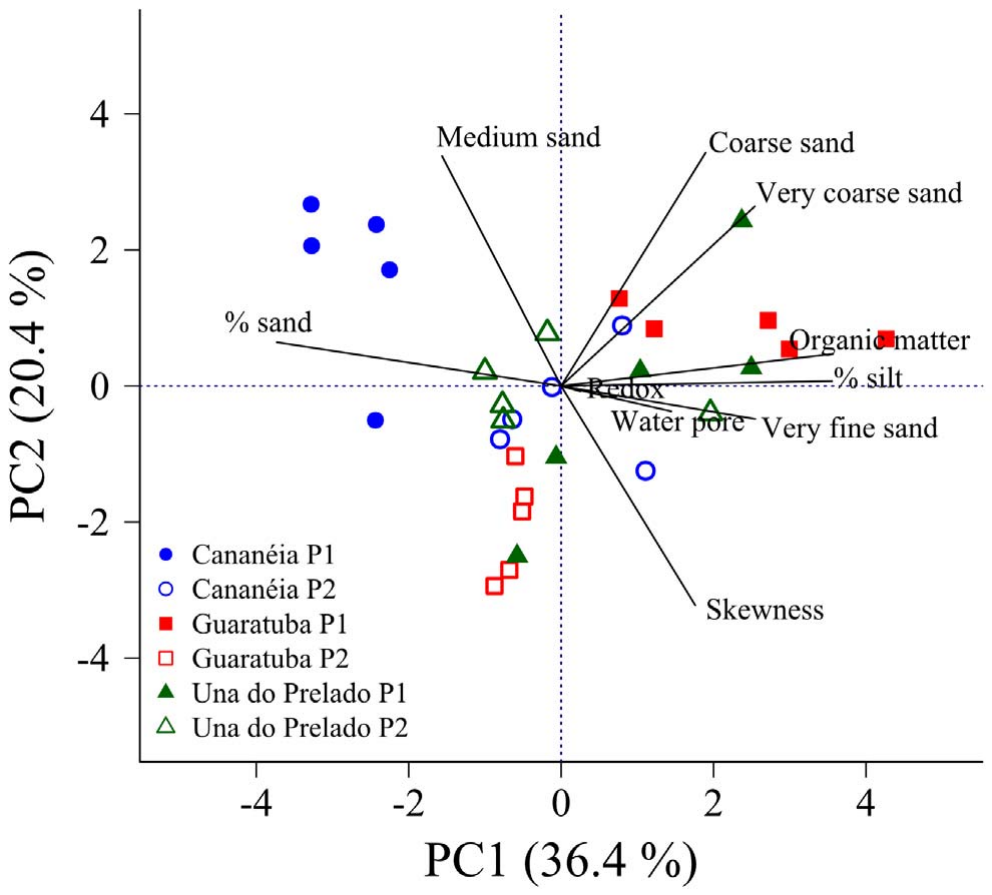
Principal component analysis. PCA of the main abiotic parameters evaluated at Guaratuba, Una do Prelado e Cananéia. Filled and empty symbols represent Plots 1 and 2, respectively.

PCA analysis on the abiotic parameters showed that PC1 and PC2 accounted for 36.4% and 20.4% of total variation present, respectively, and it showed no clustering of estuaries or layers. Data was instead clustered into plots within each estuary (Figure 3). At Guaratuba and Una do Prelado, differences between plots were mainly driven by differences in organic matter and redox potential. At Cananéia plots differed by the presence of medium sand in Plot 1 and very fine sand in Plot 2. Mean grain sizes were 2.57±0.08 φ, 2.65±0.09 φ, 2.75±0.14 φ at Cananeia, Guaratuba and Una do Prelado respectively.

### 2 - Abundance and Species Richness

ANOVA results for species richness and species diversity expressed as ES21 were practically identical (Table 2). Nematode abundance and species richness did not differ between estuaries (Table 2). Significant differences for both univariate parameters were observed for the interaction effect layers between plots (P(E)*L; Table 2). At Guaratuba differences in species richness between plots were restricted to the deepest layer, while at Cananéia significant differences were found at all depths.251657728

**Figure 4:**
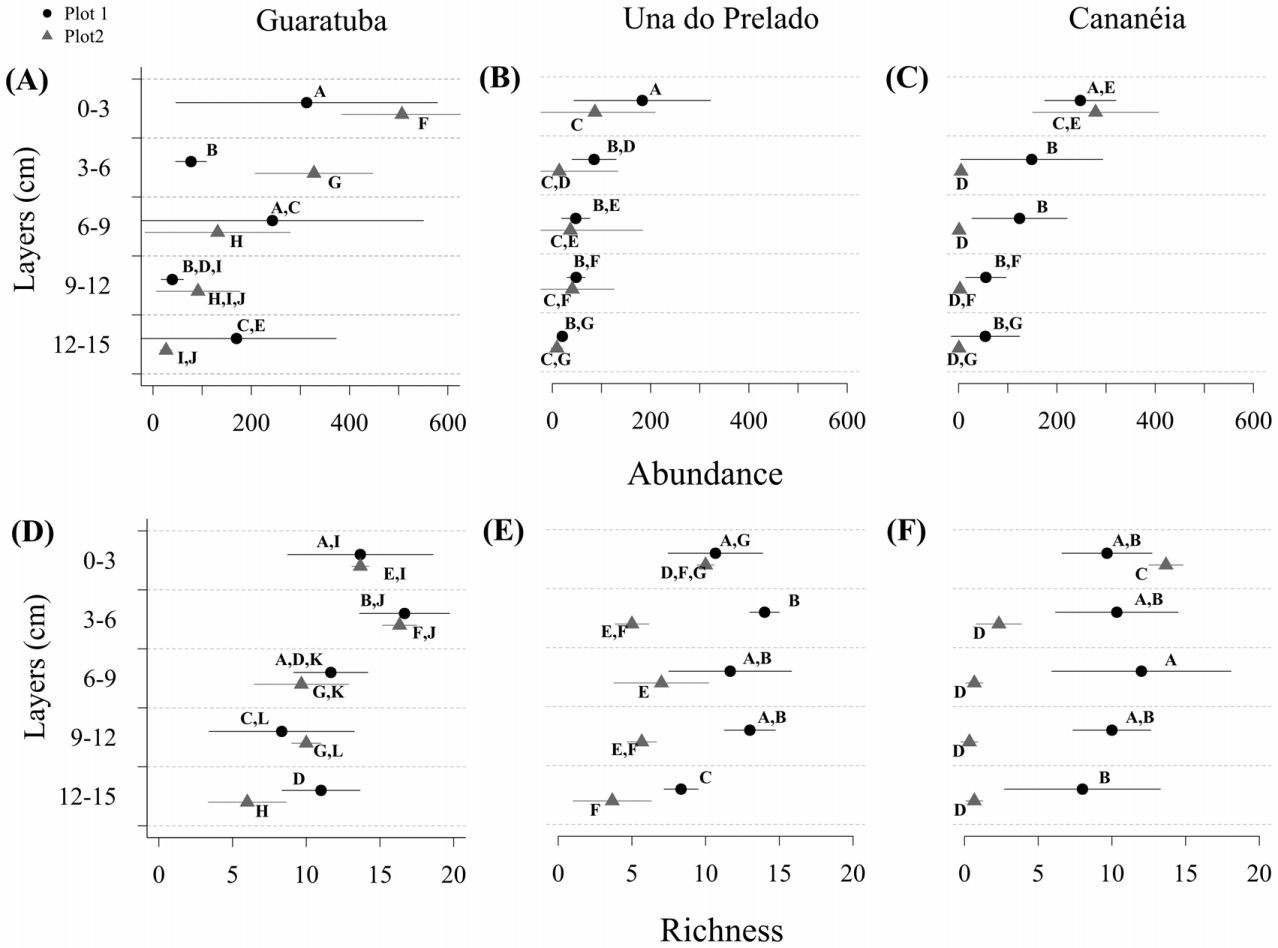
Vertical profile of univariate descriptors of the fauna. (A, B, C) Nematode abundances and (D, E, F) species richness in all sampled sites. Bars represent standard deviation from the mean (n = 3 for each point). Different letters represent significant differences found after post-hoc tests.

In general, nematode abundances and species richness were highest at the sediment surface and decreased gradually with depth (Figure 4), with the exception of Plot 1 at Guaratuba estuary where the abundance showed an alternating pattern between layers (Figure 4A). At the surface, nematode abundance varied between 52 and 900 ind. 10 cm^2^, while at the deepest layer varied from 0 to 571 ind. 10 cm^2^. Species richness varied from 7 to 17 and between 0 and 14 in the top and bottom layer, respectively.

### 3 - Community Structure

A total of 82 nematodes species were indentified, of which only two (*Desmodora cazca* and *Trochamus* sp 1) were classified as stout, the remaining were all slender. Like for the univariate measures, species composition based on presence/absence data did not differ between estuaries. Differences between plots within estuaries were dependent on the vertical layer analyzed, on the same way differences between layers were dependent on the plot (interaction effect P(E)*L; Table 2). While at Cananéia plots were significantly different from each other at all sediment layers, at Una do Prelado significant differences between plots were restricted to the deeper layers (3–15 cm) (Appendix S1).

**Figure 5:**
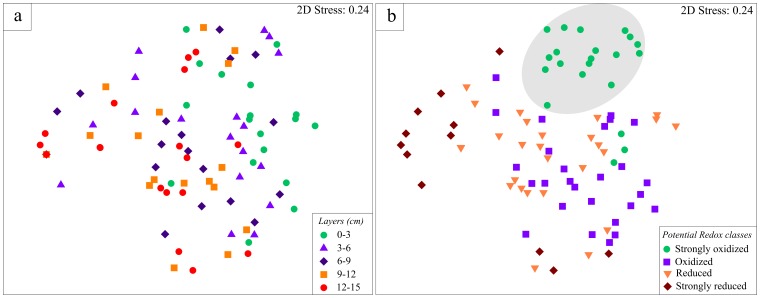
non-metric MDS ordination plot. Based on Bray-Curtis dissimilarities of presence/absence of species. (a) five sediment layers; (b) four classes of oxidation of sediment.

The MDS plot on presence/absence of nematode species showed no clustering for layers (Figure 5a). However, when this analysis was repeated using the four classes of redox potential, a cluster among samples classified as “strongly oxidized” was observed (Figure 5b). Results from SIMPER analysis based on presence/absence data revealed that the differences between “strongly oxidized” samples and the other categories were mainly due to the higher frequency of *Viscosia* sp.1, *Pomponema* sp.1, *Microlaimus* sp.4, *Cobbia* sp.1 and *Microlaimus* sp.5. (Table 3). The average similarity of the samples decreased with increasing depth. Strongly oxidized samples were about ten times more similar to each other than samples marked as “strongly reduced” (37.45% vs 3.47%).

**Table 3:** SIMPER analysis showing species ranked according to average Bray-Curtis dissimilarity between classes of potential redox.

Species	Frequency	Contrib%	Cum.%
Strongly oxidized	Average similarity: 37.45%		
*Viscosia* sp.1	0.76	13.53	13.53
*Pomponema* sp.1	0.71	11.67	25.2
*Microlaimus* sp.4	0.71	10.91	36.11
*Cobbia* sp.1	0.67	10.41	46.53
*Microlaimus* sp.5	0.67	9.51	56.03
**Oxidized**	Average similarity: 34.04%		
*Sabatieria* sp.3	0.89	20.9	20.9
*Pomponema* sp.1	0.63	11.35	32.25
*Viscosia* sp.1	0.56	8.4	40.65
*Trochamus* sp.1	0.56	8.08	48.74
*Odontophora urotrix*	0.56	7.74	56.48
**Reduced**	Average similarity: 31.38%		
*Pomponema* sp.1	0.81	27.4	27.4
*Odontophora urotrix*	0.67	16.59	43.99
*Sabatieria* sp.3	0.63	13.02	57.01
**Strongly reduced**	Average similarity: 3.47%		
*Pomponema* sp.1	0.13	19.42	19.42
*Spirinia* sp.1	0.2	18.77	38.19
*Sabatieria* sp.3	0.2	11.97	50.16

The list of species was limited to a cumulative percentage dissimilarity of 50%, i.e. when 50% of the dissimilarity was reached, remaining species were skipped. Abreviations: Contrib% - Percentage of contribution to similarity; Cum% - Cumulative percentage of contribution to similarity.

### 4 - Correlations between the Environmental Variables and the Fauna

Forward DistLM showed that redox potential was the most important variable explaining, 12% and 33% of the total variation in abundance and species richness, respectively (p<0.001). After including other environmental factors these models explained respectively 24% and 42%. (Appendix S2). Redox potential also explained 12% of the total variation observed in species composition (p<0.001); followed by organic matter, medium and very fine sand, and the percentages of silt and sand, which all together explained 35% of the fauna variability (Appendix S2).

### 5– Relationship between Community Variability, Sediment Layer and Redox Potential

**Figure 6:**
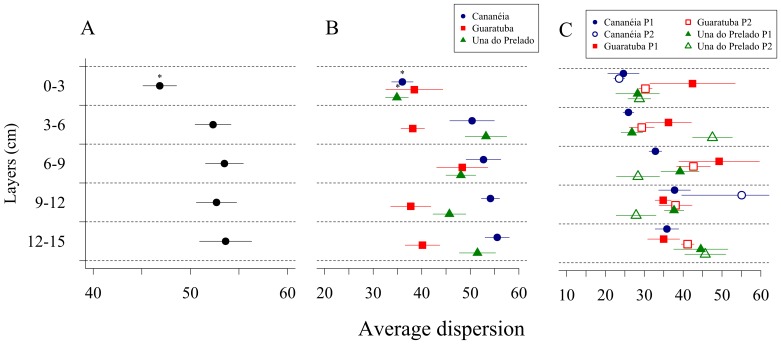
Average dispersion of the community structure. Results of PERMDISP analysis on presence-absence data along the depth gradient calculated for all estuaries together (A) and separated (B) and for each plot within estuary (C).

Dispersion of the multivariate data set was significantly lower at the first sediment layer and did not differ between the deeper layers when considering all estuaries together (Figure 6A). Although not always significant, analysis for each estuary separately also showed lower dispersion at the top most sediment layer (Figure 6B). At the smallest spatial scale (within plots), no significant differences were observed (Figure 6C). This analysis was consistent whether the data were analyzed by means of presence/absence or relative abundances. Pairwise analyzes between sediment layers within each spatial scale are listed in Appendix S3.

**Figure 7:**
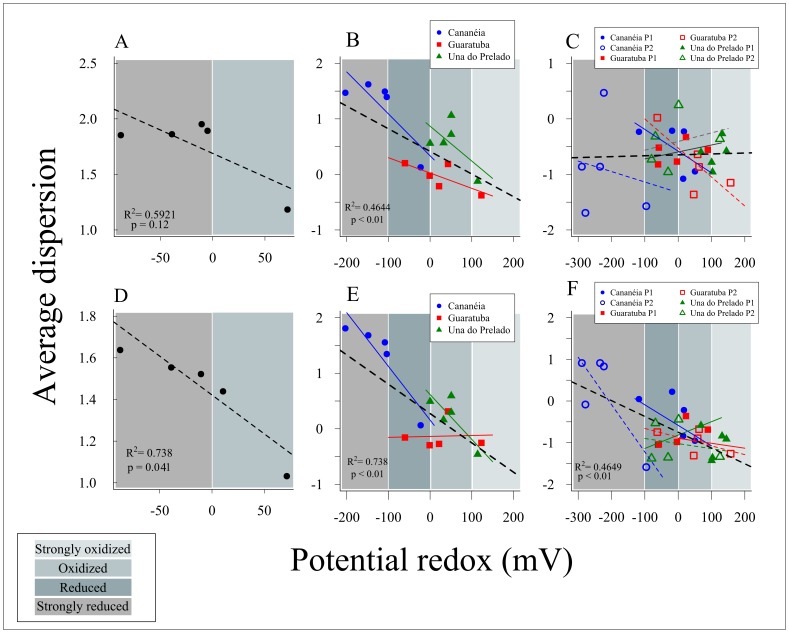
Linear regression of average multivariate dispersion against sediment redox potential. A, B and C represent results from presence/absence data; D, E, and F represent results from relative abundance data. This analysis was performed to the different sources of variation: all estuaries together (A, D) and separated (B, E), and for each plot within estuary (C, F).

Since the redox potential was the main factor selected by DistLM to explain the variability in species composition, we tested whether the dispersion of the data was related to redox values at the different spatial scales. For the presence/absence data set, dispersion was negatively related with redox only at the larger scales (Figure 7A, B, C). When analyzing the data set based on the relative abundance, the negative relationship was significant at all scales (Figure 7D, E, F).

## Discussion

The present study rejected our hypothesis that assemblages inhabiting more reduced layers of the sediment are more similar to each other at horizontal dimension. We observed that assemblages living in the top more oxygenated layers are in fact more abundant, species rich and at the same time are actually less variable, in terms of species presence/absence and relative abundances, than assemblages living in the deeper reduced layers. Although we observed species typical of reduced sediments, such as *Sabatieria* sp.3 and *Spirinia* sp.1 in samples classified as “strongly reduced”, this sediment class showed very low similarity along the horizontal scale suggesting that there is not a specific set of species living under these conditions. These findings contradict previous assumptions that the range of tolerance to extreme reduced conditions is species-specific [2], [3], [11], [13], [38]. It is important to note however that all previous studies were either experimental manipulations of a reduced set of species, or did not strictly compare the multivariate dispersion of each sediment layer.

**Figure 8:**
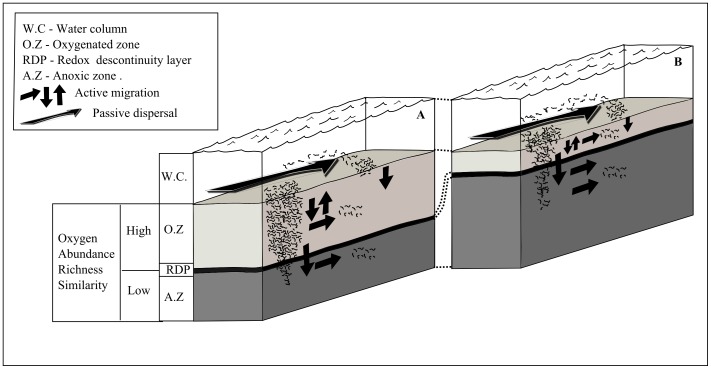
Schematic illustration. Representation of the three dimensions of the sediment matrix emphasizing potential interacting processes structuring the infauna under two hypothetical redox gradients: (A) Large oxygenated layer; (B) small oxygenated layer.

The present data also indicates that community patterns in the sediment are better explained by changes in redox potential than sediment depth *per se*. All three parameters of the fauna (abundance, species richness and community similarity) were better explained by differences in redox potentials. The importance of redox in structuring benthic communities along the vertical dimension is well known [12], however this study shows that redox can influence the fauna in both dimensions, vertical and horizontal. Basically, we can hypothesize that at the more oxygenated superficial sediment, organisms have the opportunity to colonize and/or migrate on a wide range of depths within the sediment (Figure 8). The most probable mechanism causing this pattern is that at surface layers, water current promotes passive redistribution, and the benign conditions in the sediment permits the establishment and coexistence of many species (Figure 8). There is already evidence that dispersal of nematodes occurs mainly through passive processes, via hydrodynamic forces [39]–[42]. In the deeper layers, in contrast, species are not exposed to hydrodynamism and species chiefly arrive by active migration and are therefore limited by the surrounding set of species [43]. Similar to source-sinks models [44], [45], the colonization of this layer will be mainly dependent on the rates of immigration and emigration, localized environmental conditions and species interactions. Empirical evidences supporting that the colonization processes may operate differently in superficial and deep layers comes from a series of previous experiments on meiofauna [40].

The model above would probably apply for those species which are distributed according to the redox profile [46]–[49]. Tube forming species, or species that agglutinate sediments will not fit to this model, since passive dispersion has much less importance. This model may also apply for other habitats where the sediment composition varies according to the oceanographic currents. For instance, nematode assemblages from the deep-slope (2000 m water depth) from the top layers were also more homogeneous at the horizontal scale than assemblages from the bottom layers [8]. Our dispersion model may not be applicable for stable habitats, such as abyssal plains, where water currents are generally weak to promote long distance passive dispersion. In this kind of habitat we could expect that top and deep layers are highly variable at the horizontal scale. Although our assemblage had only 2 species of stout nematodes (*Desmodora cazca* and *Trochamus* sp1), is important to note that sediments dominated by stout nematodes may show a different relationship between the vertical and horizontal scales. That is because some studies have shown that stout nematodes may be unable to vertically migrate in the sediment column [50], [51] and colonize the deeper layers. On the other hand, slender nematodes may have a greater colonization ability via infaunal migration [28], [52].

The patterns above were detected however when comparing communities at large scales, i.e. between estuaries and plots within estuaries. At the smaller scale, within plots, the increasing variability in species composition with increasing redox potential or sediment depth was much less evident. The negative relationship was only detected on the relative abundance data set, suggesting that community changes at the small scale are more subtle and not perceived with presence/absence transformation. At the small horizontal scale there was little species replacement and most of the species occurred with different relative abundances. The lack of patterns observed within plots could be a consequence of local factors as relation predator-prey [53] and presence of macrofaunal species [54], [55], since these organisms have a important role to sediment dynamics in the recovery and structuring of nematodes communities [56]. Besides, meiofaunal organisms can benefit from macrobenthic engineering presence leading typical surface organisms to inhabit surroundings of the burrows at deeper layers of the sediment [57]. Probably at this small scale biotic interactions [53], [58], [59] and stochasticity are more important in structuring the fauna than redox potential alone. Small-scale variability in nematode composition is in fact less predictable than at larger scales [6], [8], [60]. However, is important to emphasize that the low number of replicates at plot scale (n = 2) might not have been enough to adequately characterize this spatial level.

Although the current community patterns at both horizontal and vertical dimensions were mainly driven by redox and just weakly explained by organic matter content, one cannot exclude the possibility of the role of food quality [61], [62]. Especially in food limited environment, like the deep sea, food quality is known to drive vertical and horizontal patterns of the meiofauna [63]. However, evidence for highly productive areas like estuaries is still inconclusive [1].

Another important fact to be discussed is the continuous decrease in nematode abundances and species richness with increasing depth in the sediment. Significant differences were restricted between the uppermost (0–3 cm) and deepest layer (12–15 cm), the intermediate layers (3–6, 6–9 and 9–12 cm) were highly variable and did not differ from each other. This high variability could be due to the inherent characteristics of estuarine bedforms, since they are highly dynamic environments, and/or a consequence of the independent sampling design adopted. It is well accepted that vertical distribution within the sediment are determined by environmental gradients [16], [33], [64] and biological interactions [9], [53], [65], [66]. As such, studies using a dependent sampling design [e.g. 2,13,14,19], would artificially reinforce differences between layers because of the reduced variability sampled. The independent sampling design used in the present study better characterizes the variability between replicates and thus the spatial patterns of the fauna. By using such design the vertical changes in communities won’t be as evident as previously expected. We strongly recommend that an independent sampling design should be adopted if horizontal and vertical patterns are intended to be investigated simultaneously.

## Supporting Information

Appendix S1 **Pairwise comparisons of PERMANOVA comparison.** Pairwise tests P-value based on Monte Carlo (MC) of community structure for different sources of variation. Bold lettering identifies those P-values that are significant (<0.05). L1, L2, L3, L4 and L5.
(DOC)

Appendix S2 **Results of Distance-based multivariate analysis for a linear model (DistLM).** Results of forward distance-based multivariate analysis for a linear model (DistLM). SS = sum of squares; F = pseudo-F; P = p- value; Prop = proportion of explanation; Cumul = Cumulative proportion of explanation; res.df = residual degree of freedom.
(DOC)

Appendix S3 **Pairwise comparisons of PERMDISP.** Pairwise tests of Permutational analysis of multivariate dispersions (PERMDISP) under presence/absence species of nematodes at different sources of variation. Bold lettering identifies those P-values that are significant (<0.05). L1, L2, L3, L4 and L5 correspond respectively to vertical strata 0–3, 3–6, 6–9, 9–12, 12–15 cm.
(DOC)
