# Supplementary material for: The Importance of Vertical and Horizontal Dimensions of the Sediment Matrix in Structuring Nematodes Across Spatial Scales
Source: PLoS One. 2013 Oct 30;8(10):e77704. doi: 10.1371/journal.pone.0077704 (PMC3813771; doi:10.1371/journal.pone.0077704)
Supplement: Appendix S1 — Pairwise comparisons of PERMANOVA comparison. Pairwise tests P-value based on Monte Carlo (MC) of community structure for different sources of variation. Bold lettering identifies those P-values that are significant (<0.05). L1, L2, L3, L4 and L5. (DOC) [file pone.0077704.s001.doc]

Supporting Information

**Appendix S1.**

| Source of Variation | Comparison level | Group | t | P(MC) |
| --- | --- | --- | --- | --- |
| Layer | All estuaries together | L1, L2 | 1.6197 | 0.063 |
|  |  | L1, L3 | 1.7627 | 0.066 |
|  |  | L1, L4 | 2.0495 | **0.035** |
|  |  | L1, L5 | 2.0654 | **0.022** |
|  |  | L2, L3 | 1.4249 | 0.123 |
|  |  | L2, L4 | 1.899 | **0.024** |
|  |  | L2, L5 | 1.8444 | **0.026** |
|  |  | L3, L4 | 1.1676 | 0.313 |
|  |  | L3, L5 | 1.7517 | **0.024** |
|  |  | L4, L5 | 1.4419 | 0.126 |
| Plot(Estuary) | Cananéia | P1, P2 | 5.1617 | **0.001** |
|  | Guaratuba | P1, P2 | 2.9881 | **0.001** |
|  | Una do Prelado | P1, P2 | 3.9271 | **0.001** |
| Plot(Estuary)*Layer | Cananéia P1 | L1, L2 | 1.1862 | 0.299 |
|  |  | L1, L3 | 1.4741 | 0.112 |
|  |  | L1, L4 | 1.912 | 0.098 |
|  |  | L1, L5 | 1.6095 | **0.095** |
|  |  | L2, L3 | 1.1727 | 0.372 |
|  |  | L2, L4 | 1.611 | 0.1 |
|  |  | L2, L5 | 1.3463 | 0.394 |
|  |  | L3, L4 | 0.8014 | 0.778 |
|  |  | L3, L5 | 0.93413 | 0.52 |
|  |  | L4, L5 | 0.83971 | 0.596 |
|  | Cananéia P2 | L1, L2 | 2.1351 | 0.101 |
|  |  | L1, L3 | 3.7498 | 0.118 |
|  |  | L1, L4 | 5.0858 | 0.081 |
|  |  | L1, L5 | 3.9643 | 0.115 |
|  |  | L2, L3 | 1.2024 | 0.293 |
|  |  | L2, L4 | 1.2769 | 0.315 |
|  |  | L2, L5 | 1.2024 | 0.292 |
|  |  | L3, L4 | 1.0198 | 0.908 |
|  |  | L3, L5 | 1.029 | 0.602 |
|  |  | L4, L5 | 1.0198 | 0.901 |
|  | Guaratuba P1 | L1, L2 | 1.3675 | 0.192 |
|  |  | L1, L3 | 1.2179 | 0.2 |
|  |  | L1, L4 | 1.8769 | 0.095 |
|  |  | L1, L5 | 1.3553 | 0.197 |
|  |  | L2, L3 | 1.0718 | 0.433 |
|  |  | L2, L4 | 2.0206 | 0.094 |
|  |  | L2, L5 | 1.1967 | 0.426 |
|  |  | L3, L4 | 1.2295 | 0.192 |
|  |  | L3, L5 | 0.68901 | 0.787 |
|  |  | L4, L5 | 0.8508 | 0.597 |
|  | Guaratuba P2 | L1, L2 | 1.1778 | 0.205 |
|  |  | L1, L3 | 1.8719 | 0.104 |
|  |  | L1, L4 | 1.9792 | 0.101 |
|  |  | L1, L5 | 2.0077 | 0.106 |
|  |  | L2, L3 | 1.3323 | 0.103 |
|  |  | L2, L4 | 1.5463 | 0.103 |
|  |  | L2, L5 | 1.7178 | 0.095 |
|  |  | L3, L4 | 1.3009 | 0.18 |
|  |  | L3, L5 | 1.1846 | 0.293 |
|  |  | L4, L5 | 1.2833 | 0.103 |
|  | Una do Prelado P1 | L1, L2 | 1.9251 | 0.115 |

*Appendix S1 continued*

| Source of Variation | Comparison level | Group | t | P(MC) |
| --- | --- | --- | --- | --- |
| Plot(Estuary)*Layer |  | L1, L3 | 1.816 | 0.097 |
|  |  | L1, L4 | 2.2869 | 0.092 |
|  |  | L1, L5 | 2.1055 | 0.091 |
|  |  | L2, L3 | 1.004 | 0.594 |
|  |  | L2, L4 | 1.6133 | 0.11 |
|  |  | L2, L5 | 2.0603 | 0.088 |
|  |  | L3, L4 | 0.99405 | 0.718 |
|  |  | L3, L5 | 1.3865 | 0.19 |
|  |  | L4, L5 | 1.2572 | 0.183 |
|  | Una do Prelado P2 | L1, L2 | 1.1764 | 0.205 |
|  |  | L1, L3 | 2.1521 | 0.095 |
|  |  | L1, L4 | 2.5785 | 0.096 |
|  |  | L1, L5 | 2.0427 | 0.11 |
|  |  | L2, L3 | 1.3699 | 0.119 |
|  |  | L2, L4 | 1.43 | 0.094 |
|  |  | L2, L5 | 1.0915 | 0.311 |
|  |  | L3, L4 | 1.2023 | 0.295 |
|  |  | L3, L5 | 1.3892 | 0.311 |
|  |  | L4, L5 | 1.3667 | 0.403 |
| Plot(Estuary)*Layer | Cananéia L1 | P1,P2 | 2.5414 | **0.015** |
|  | Cananéia L2 | P1,P2 | 2.2437 | **0.022** |
|  | Cananéia L3 | P1,P2 | 2.7413 | **0.02** |
|  | Cananéia L4 | P1,P2 | 3.3081 | **0.006** |
|  | Cananéia L5 | P1,P2 | 2.5861 | **0.019** |
|  | Guaratuba L1 | P1,P2 | 1.4098 | 0.156 |
|  | Guaratuba L2 | P1, P2 | 2.0296 | **0.031** |
|  | Guaratuba L3 | P1, P2 | 1.8405 | 0.052 |
|  | Guaratuba L4 | P1, P2 | 1.7225 | 0.09 |
|  | Guaratuba L5 | P1, P2 | 1.3976 | 0.153 |
|  | Una do Prelado L1 | P1, P2 | 1.4812 | 0.129 |
|  | Una do Prelado L2 | P1, P2 | 2.3121 | **0.019** |
|  | Una do Prelado L3 | P1, P2 | 2.4354 | **0.02** |
|  | Una do Prelado L4 | P1, P2 | 2.399 | **0.022** |
|  | Una do Prelado L5 | P1, P2 | 2.0304 | **0.026** |
