# Supplementary material for: The Importance of Vertical and Horizontal Dimensions of the Sediment Matrix in Structuring Nematodes Across Spatial Scales
Source: PLoS One. 2013 Oct 30;8(10):e77704. doi: 10.1371/journal.pone.0077704 (PMC3813771; doi:10.1371/journal.pone.0077704)
Supplement: Appendix S2 — Results of Distance-based multivariate analysis for a linear model (DistLM). Results of forward distance-based multivariate analysis for a linear model (DistLM). SS = sum of squares; F = pseudo-F; P = p- value; Prop = proportion of explanation; Cumul = Cumulative proportion of explanation; res.df = residual degree of freedom. (DOC) [file pone.0077704.s002.doc]

Supporting Information

**Appendix S2**

|  | Variable | Adj R^2^ | SS | F | P | Prop. | Cumul. | res.df |
| --- | --- | --- | --- | --- | --- | --- | --- | --- |
| *Abundance* | Redox | 0.115 | 250100 | 12.58 | **0.00** | 0.13 | 0.13 | 88 |
|  | % Silt | 0.141 | 70164 | 3.64 | **0.05** | 0.04 | 0.16 | 87 |
|  | Very fine sand | 0.188 | 110220 | 6.04 | **0.02** | 0.06 | 0.22 | 86 |
|  | Assymetry | 0.197 | 36214 | 2.01 | 0.17 | 0.02 | 0.23 | 85 |
|  | Medium sand | 0.202 | 26919 | 1.5 | 0.22 | 0.01 | 0.25 | 84 |
| *Richness* | Redox | 0.326 | 752.5 | 44.05 | **0.00** | 0.33 | 0.33 | 88 |
|  | % Silt | 0.346 | 60.361 | 3.64 | 0.08 | 0.03 | 0.36 | 87 |
|  | % Sand | 0.378 | 87.431 | 5.55 | **0.02** | 0.04 | 0.4 | 86 |
| *Presence/absence* | Redox | 0.111 | 26139 | 12.06 | **0.00** | 0.12 | 0.12 | 88 |
|  | Organic Matter | 0.188 | 18539 | 9.37 | **0.00** | 0.09 | 0.21 | 87 |
|  | Medium sand | 0.243 | 13524 | 7.33 | **0.00** | 0.06 | 0.27 | 86 |
|  | % Silt | 0.261 | 5540.6 | 3.08 | **0.00** | 0.03 | 0.29 | 85 |
|  | % Sand | 0.297 | 9294.5 | 5.43 | **0.00** | 0.04 | 0.34 | 84 |
|  | Very fine sand | 0.304 | 3082.8 | 1.82 | 0.07 | 0.01 | 0.35 | 83 |
|  | Assymetry | 0.309 | 2586.2 | 1.54 | 0.13 | 0.01 | 0.36 | 82 |
|  | Water pore | 0.311 | 2098.8 | 1.25 | 0.24 | 0.01 | 0.37 | 81 |
| *Presence/absence for layer* |  |  |  |  |  |  |  |  |
| Layer 1 | Medium sand | 0.187 | 9501.8 | 4.92 | **0.00** | 0.24 | 0.24 | 16 |
|  | % silt | 0.289 | 5548.8 | 3.28 | **0.01** | 0.14 | 0.37 | 15 |
|  | Organic Matter | 0.431 | 6423.4 | 4.74 | **0.00** | 0.16 | 0.53 | 14 |
|  | Assymetry | 0.551 | 5086.2 | 4.77 | **0.00** | 0.13 | 0.66 | 13 |
|  | Very fine sand | 0.649 | 3856.7 | 4.62 | **0.00** | 0.1 | 0.75 | 12 |
|  | Redox | 0.654 | 956.48 | 1.16 | 0.34 | 0.02 | 0.78 | 11 |
|  | Water pore | 0.654 | 0 | 0 | 1 | 0 | 0.78 | 11 |
| Layer 2 | Medium sand | 0.127 | 8957.7 | 3.46 | **0.00** | 0.18 | 0.18 | 16 |
|  | Water pore | 0.25 | 8060.6 | 3.63 | **0.00** | 0.16 | 0.34 | 15 |
|  | Organic Matter | 0.348 | 6283.4 | 3.25 | **0.00** | 0.12 | 0.46 | 14 |
|  | Assymetry | 0.418 | 4624.1 | 2.68 | **0.01** | 0.09 | 0.55 | 13 |
|  | % sand | 0.517 | 5247 | 3.67 | **0.00** | 0.1 | 0.66 | 12 |
|  | Redox | 0.518 | 1478.8 | 1.04 | 0.41 | 0.03 | 0.69 | 11 |
|  | Very fine sand | 0.517 | 5247 | 3.67 | **0.00** | 0.1 | 0.66 | 12 |
| Layer 3 | Medium sand | 0.1 | 8041.1 | 2.88 | **0.00** | 0.15 | 0.15 | 16 |
|  | Very fine sand | 0.194 | 7200 | 2.88 | **0.00** | 0.14 | 0.29 | 15 |
|  | Assymetry | 0.293 | 6773.5 | 3.09 | **0.00** | 0.13 | 0.42 | 14 |
|  | % silt | 0.371 | 5355.7 | 2.75 | **0.01** | 0.1 | 0.52 | 13 |
|  | Water pore | 0.445 | 4699.8 | 2.73 | **0.00** | 0.09 | 0.61 | 12 |
|  | Redox | 0.492 | 3311.6 | 2.1 | **0.02** | 0.06 | 0.67 | 11 |
|  | Organic Matter | 0.492 | 0 | 0 | 1 | 0 | 0.67 | 11 |
| Layer 4 | Very fine sand | 0.101 | 7914.4 | 2.92 | **0.00** | 0.15 | 0.15 | 16 |
|  | Redox | 0.205 | 7388.5 | 3.08 | **0.00** | 0.14 | 0.3 | 15 |
|  | Water pore | 0.319 | 7239.1 | 3.53 | **0.00** | 0.14 | 0.44 | 14 |
|  | Assymetry | 0.493 | 8861.9 | 5.79 | **0.00** | 0.17 | 0.61 | 13 |
|  | Medium sand | 0.52 | 2494.8 | 1.72 | 0.12 | 0.05 | 0.66 | 12 |
|  | Organic Matter | 0.549 | 2428.4 | 1.79 | 0.1 | 0.05 | 0.71 | 11 |
| Layer 5 | Very fine sand | 0.114 | 8955 | 3.18 | **0.00** | 0.17 | 0.17 | 16 |
|  | Assymetry | 0.194 | 6625.7 | 2.59 | **0.01** | 0.12 | 0.29 | 15 |
|  | Redox | 0.276 | 6196.1 | 2.69 | **0.01** | 0.11 | 0.4 | 14 |
|  | % silt | 0.346 | 5206.7 | 2.51 | **0.01** | 0.1 | 0.5 | 13 |
|  | % sand | 0.371 | 3023.6 | 1.51 | 0.09 | 0.06 | 0.56 | 12 |
|  | Organic Matter | 0.427 | 3937.8 | 2.16 | **0.00** | 0.07 | 0.63 | 11 |
|  | Medium sand | 0.427 | 6727.7 | 3.7 | **0.00** | 0.12 | 0.63 | 11 |
