# Supplementary material for: The Importance of Vertical and Horizontal Dimensions of the Sediment Matrix in Structuring Nematodes Across Spatial Scales
Source: PLoS One. 2013 Oct 30;8(10):e77704. doi: 10.1371/journal.pone.0077704 (PMC3813771; doi:10.1371/journal.pone.0077704)
Supplement: Appendix S3 — Pairwise comparisons of PERMDISP. Pairwise tests of Permutational analysis of multivariate dispersions (PERMDISP) under presence/absence species of nematodes at different sources of variation. Bold lettering identifies those P-values that are significant (<0.05). L1, L2, L3, L4 and L5 correspond respectively to vertical strata 0–3, 3–6, 6–9, 9–12, 12–15 cm. (DOC) [file pone.0077704.s003.doc]

Supporting Information

**Appendix S3**

| Source of Variation | Comparasion level | Group | T | P |
| --- | --- | --- | --- | --- |
| Layer | All estuaries together | L1,L2 | 2.88 | **0.01** |
|  |  | L1,L3 | 2.58 | **0.01** |
|  |  | L1,L4 | 2.54 | **0.01** |
|  |  | L1,L5 | 2.43 | **0.04** |
|  |  | L2,L3 | 0.23 | 0.86 |
|  |  | L2,L4 | 0.36 | 0.74 |
|  |  | L2,L5 | 0.37 | 0.72 |
|  |  | L3,L4 | 0.12 | 0.92 |
|  |  | L3,L5 | 0.14 | 0.89 |
|  |  | L4,L5 | 0.03 | 0.98 |
| Estuary*layer | Cananéia | L1,L2 | 3.15 | **0.01** |
|  |  | L1,L3 | 4.10 | **0.00** |
|  |  | L1,L4 | 6.19 | **0.00** |
|  |  | L1,L5 | 4.13 | **0.00** |
|  |  | L2,L3 | 0.20 | 0.83 |
|  |  | L2,L4 | 0.52 | 0.55 |
|  |  | L2,L5 | 0.16 | 0.89 |
|  |  | L3,L4 | 0.35 | 0.69 |
|  |  | L3,L5 | 0.05 | 0.96 |
|  |  | L4,L5 | 0.41 | 0.61 |
|  | Guaratuba | L1,L2 | 0.46 | 0.69 |
|  |  | L1,L3 | 1.25 | 0.25 |
|  |  | L1,L4 | 1.03 | 0.41 |
|  |  | L1,L5 | 1.43 | 0.24 |
|  |  | L2,L3 | 1.01 | 0.38 |
|  |  | L2,L4 | 0.70 | 0.50 |
|  |  | L2,L5 | 1.21 | 0.27 |
|  |  | L3,L4 | 0.55 | 0.64 |
|  |  | L3,L5 | 0.03 | 0.99 |
|  |  | L4,L5 | 0.70 | 0.55 |
|  | Una do Prelado | L1,L2 | 3.14 | **0.02** |
|  |  | L1,L3 | 2.47 | **0.03** |
|  |  | L1,L4 | 2.00 | 0.07 |
|  |  | L1,L5 | 1.75 | 0.11 |
|  |  | L2,L3 | 0.90 | 0.38 |
|  |  | L2,L4 | 1.28 | 0.22 |
|  |  | L2,L5 | 1.19 | 0.26 |
|  |  | L3,L4 | 0.43 | 0.63 |
|  |  | L3,L5 | 0.41 | 0.66 |
|  |  | L4,L5 | 0.02 | 0.98 |
| Plot(Estuary)*Layer | Cananéia L1 | P1,P2 | 1.43 | 0.30 |
|  | Cananéia L2 | P1,P2 | 2.37 | 0.10 |
|  | Cananéia L3 | P1,P2 | 1.07 | 0.72 |
|  | Cananéia L4 | P1,P2 | 2.36 | 0.40 |
|  | Cananéia L5 | P1,P2 | 0.66 | 0.80 |
|  | Guaratuba L1 | P1,P2 | 0.93 | 0.90 |
|  | Guaratuba L2 | P1, P2 | 1.84 | 0.21 |
|  | Guaratuba L3 | P1, P2 | 0.93 | 0.59 |
|  | Guaratuba L4 | P1, P2 | 0.37 | 0.81 |
|  | Guaratuba L5 | P1, P2 | 1.57 | 0.30 |
|  | Una do Prelado L1 | P1, P2 | 1.15 | 0.53 |
|  | Una do Prelado L2 | P1, P2 | 2.47 | 0.11 |
|  | Una do Prelado L3 | P1, P2 | 1.53 | 0.20 |

*Appendix S*3 continued.

| Source of Variation | Comparasion level | Group | T | P |
| --- | --- | --- | --- | --- |
| Plot(Estuary)*Layer | Una do Prelado L4 | P1, P2 | 0.53 | 0.71 |
|  | Una do Prelado L5 | P1, P2 | 0.40 | 0.90 |
|  | Cananéia P1 | L1, L2 | 0.36 | 0.62 |
|  |  | L1, L3 | 1.42 | 0.59 |
|  |  | L1, L4 | 1.32 | 0.50 |
|  |  | L1, L5 | 0.79 | 0.80 |
|  |  | L2, L3 | 1.80 | 0.12 |
|  |  | L2, L4 | 1.65 | 0.32 |
|  |  | L2, L5 | 0.95 | 0.50 |
|  |  | L3, L4 | 0.02 | 1.00 |
|  |  | L3, L5 | 0.01 | 1.00 |
|  |  | L4, L5 | 0.02 | 1.00 |
|  | Cananéia P2 | L1, L2 | 2.95 | 0.11 |
|  |  | L1, L3 | 1.34 | 0.40 |
|  |  | L1, L4 | 0.23 | 0.61 |
|  |  | L1, L5 | 1.34 | 0.38 |
|  |  | L2, L3 | 1.79 | 0.22 |
|  |  | L2, L4 | 2.93 | 0.10 |
|  |  | L2, L5 | 1.79 | 0.19 |
|  |  | L3, L4 | 1.41 | 0.40 |
|  |  | L3, L5 | 0.00 | 0.76 |
|  |  | L4, L5 | 1.41 | 0.39 |
|  | Guaratuba P1 | L1, L2 | 1.22 | 0.59 |
|  |  | L1, L3 | 0.43 | 0.81 |
|  |  | L1, L4 | 0.10 | 0.89 |
|  |  | L1, L5 | 0.92 | 0.49 |
|  |  | L2, L3 | 0.85 | 0.62 |
|  |  | L2, L4 | 0.18 | 0.81 |
|  |  | L2, L5 | 0.61 | 0.48 |
|  |  | L3, L4 | 1.03 | 0.89 |
|  |  | L3, L5 | 0.33 | 0.70 |
|  |  | L4, L5 | 0.84 | 0.68 |
|  | Guaratuba P2 | L1, L2 | 0.84 | 0.52 |
|  |  | L1, L3 | 0.75 | 0.62 |
|  |  | L1, L4 | 1.05 | 0.40 |
|  |  | L1, L5 | 7.59 | 0.10 |
|  |  | L2, L3 | 1.21 | 0.51 |
|  |  | L2, L4 | 1.41 | 0.29 |
|  |  | L2, L5 | 6.15 | 0.10 |
|  |  | L3, L4 | 0.39 | 1.00 |
|  |  | L3, L5 | 2.46 | 0.09 |
|  |  | L4, L5 | 1.39 | 0.50 |
|  | Una do Prelado P1 | L1, L2 | 0.41 | 0.71 |
|  |  | L1, L3 | 1.46 | 0.42 |
|  |  | L1, L4 | 1.14 | 0.49 |
|  |  | L1, L5 | 0.87 | 0.50 |
|  |  | L2, L3 | 1.05 | 0.49 |
|  |  | L2, L4 | 0.56 | 0.70 |
|  |  | L2, L5 | 0.42 | 0.79 |
|  |  | L3, L4 | 0.74 | 0.59 |
|  |  | L3, L5 | 0.68 | 0.72 |
|  |  | L4, L5 | 0.06 | 1.00 |
|  | Una do Prelado P2 | L1, L2 | 1.18 | 0.29 |
|  |  | L1, L3 | 1.20 | 0.40 |
|  |  | L1, L4 | 0.76 | 0.63 |
|  |  | L1, L5 | 0.06 | 1.00 |
|  |  | L2, L3 | 3.28 | 0.10 |
|  |  | L2, L4 | 2.76 | 0.09 |
|  |  | L2, L5 | 0.81 | 0.73 |
|  |  | L3, L4 | 0.69 | 0.49 |
|  |  | L3, L5 | 0.94 | 0.74 |
|  |  | L4, L5 | 0.61 | 0.69 |
